# Positioning of Chromosomes in Human Spermatozoa Is Determined by Ordered Centromere Arrangement

**DOI:** 10.1371/journal.pone.0052944

**Published:** 2012-12-27

**Authors:** Olga S. Mudrak, Igor B. Nazarov, Estella L. Jones, Andrei O. Zalensky

**Affiliations:** 1 Institute of Cytology, Russian Academy of Sciences, St. Petersburg, Russia; 2 The Jones Institute for Reproductive Medicine, Eastern Virginia Medical School, Norfolk, Virginia, United States of America; University of Florence, Italy

## Abstract

The intranuclear positioning of chromosomes (CHRs) is a well-documented fact; however, mechanisms directing such ordering remain unclear. Unlike somatic cells, human spermatozoa contain distinct spatial markers and have asymmetric nuclei which make them a unique model for localizing CHR territories and matching peri-centromere domains. In this study, we established statistically preferential longitudinal and lateral positioning for eight CHRs. Both parameters demonstrated a correlation with the CHR gene densities but not with their sizes. Intranuclear non-random positioning of the CHRs was found to be driven by a specific linear order of centromeres physically interconnected in continuous arrays. In diploid spermatozoa, linear order of peri-centromeres was identical in two genome sets and essentially matched the arrangement established for haploid cells. We propose that the non-random longitudinal order of CHRs in human spermatozoa is generated during meiotic stages of spermatogenesis. The specific arrangement of sperm CHRs may serve as an epigenetic basis for differential transcription/replication and direct spatial CHR organization during early embryogenesis.

## Introduction

In higher eukaryotes, CHRs retain their distinctiveness throughout the cell cycle; in interphase they occupy well-defined nuclear sub-volumes called chromosome territories (CTs), reviewed in [1], [2], [3]. Individual CTs are characterized by the preferred intranuclear positions [4], [5], [6]. It was found that within the spherical interphase nuclei, where only the radial CHR position may be determined, the gene-poor CHRs tend to locate peripherally while the gene-rich - more centrally [7], [8], [9]. Therefore, a functional link between distribution of CTs within the nuclear space and the regulation of gene expression has been suggested [3], [10], however this association is not universal [11]. Thus, small CHRs, independent of their gene density, are located significantly closer to the nucleus center in human fibroblasts [6], [12] and amniotic fluid cells [6].

What determines a preferred CHR positioning? Is it preserved through the cell cycle and inherited? These related issues are largely unresolved and existing views are controversial. Studies on CHR arrangement in the prometaphase ring reported the presence [13] or the absence [6], [14] of a defined order. Gerlich and co-authors using rat kidney and HeLa cells demonstrated that global CHR positions are heritable through the cell cycle [15]. In other studies, major changes in CT neighborhoods from one cell cycle to the next one were shown in HeLa [16] and Retina pigment epithelium 1 cells [17]. Mechanisms that underlay a specific intranuclear localization of CTs, if such exists, are still not ascertained [9]. Human CHRs introduced into a mouse nucleus conserve “donor-specific” CHR positioning in the host cells indicating the presence of an unidentified determinant of the intranuclear CHR localization [18]. There is evidence indicating the involvement of nuclear lamins A and B1 [19], [20]. On the other hand, the mathematical modeling showed that nonrandom positions of CTs within nuclei may be warranted by the entropy driven organization of self-avoiding polymers [21].

Chromatin organization in spermatozoa differs significantly from that in somatic cells. In mammalian sperm nuclei, DNA is tightly packed with protamines and is genetically inert, reviewed in [22], [23]. At the same time, the territorial organization and non-random intranuclear arrangement of CHRs are preserved, reviewed in [24]. In this work, we explore the unique features of human spermatozoa (Hsp) (haploid set of CHRs, asymmetrical flattened nuclei, the existence of spatial markers) to investigate in detail the phenomenon of CHR positioning. We show that the preferred localization of CTs is tightly linked with the positioning of corresponding peri-centromere (peri-CEN) domains. The latter demonstrates a specific linear order within continuous centromere (CEN) arrays forming sperm chromocenter. Apparently, the pattern of CEN localization (i.e. CHR order in sperm cells) is preserved among the human population and is established during meiosis. Importantly, gene-rich CHR in Hsp tend to localize closer to the nucleus interior (in parallel with somatic cells) and towards the apical, acrosomal end of the elongated nucleus (sperm-specific feature).

## Materials and Methods

### Preparation of sperm cells for microscopy

Semen samples were obtained from normozoospermic (WHO1999) healthy men enrolled in the donor sperm program and signed consents for the use of their semen samples for various assisted conception methods as well as for research at The Jones Institute for Reproductive Medicine. The study was approved by the Institutional Review Board of Eastern Virginia Medical School and written informed consent was obtained from all participants. In all experiments a mixture of equal numbers of sperm cells from three individuals was used. Isolation of sperm cells was described earlier [25], [26]. Briefly, purified cells were fixed in 0.5% paraformaldehyde in PBS for 2 min before further steps. Before performing fluorescence in situ hybridization (FISH), sperm chromatin was mildly decondensed by the treatment with 1 mM DTT/0.05 mg/ml Heparin. The validity of such sperm cell pretreatment for the study of nuclear architecture has been demonstrated in our previous studies: a) atomic force microscopy of native human sperm nuclei vs. sperm nuclei subjected to swelling followed by several rounds of ethanol dehydration/rehydration has shown the preservation of overall nuclear morphology with uniformly increased ***x***, ***y*** dimensions and characteristic ***z***-dimension profile restored in water-based mounting media [27]; b) DTT/Heparin treatment has been shown to lead to chromatin relaxation and uniform swelling of the sperm head, yet does not cause a noticeable loss of nuclear proteins and allows the efficient and reproducible in situ hybridization of various DNA probes in sperm nuclei [25], [27]. Spermatozoa were loaded onto microscope slides, dehydrated and air-dried before mounting.

### FISH probes

Whole chromosome painting (WCP) probes were made from DNA of flow-sorted human CHRs 1, 3, 4, 6, 17, 18, 19, X and Y kindly provided by Dr. I. Solovei (Ludwig-Maximilians University, Munich, Germany). Degenerate-oligonuicleotide-primed PCR [28] was used for amplification and labeling of chromosomal DNA with digoxigenin (DIG) or fluorescein isothiocyanate (FITC) using corresponding PCR labeling kits (Roche). Directly labeled oligonucleotide pan-CEN (human alpha-satellite consensus) and CHR-specific peri-CEN probes were from Cellay, Inc.

### FISH

Standard FISH procedure with WCP probes was used. Microscope slides loaded with minimally decondensed sperm cells were denatured in 70% formamide/2xSSC for 3 min at 72°C, fixed/dehydrated in cold EtOH and air-dried. WCP probes were denatured at 72°C for 10 min, preannealed for 30 min at 37°C and applied to the slide. Overnight hybridization at 37°C was followed by post-hybridization washings in 50% formamide/2XSSC at 46°C and in 4XSSC/0.2% Tween 20 at 46°C. The cells were blocked in 5% BSA, 2XSSC, 0.1% Tween-20 for 30 min at room temperature (RT). Signals from DIG-labeled probes were detected using anti-DIG-FITC or anti -DIG-Rhodamine (Roche Diagnostics), FITC labeled probes were amplified using mouse anti-FITC (Millipore) followed by anti-Mouse-FITC (Invitrogen, Life Technologies). Slides were mounted using Vectashield mounting medium with DAPI (diamidino-2-phenylindole) (Vector Labs Inc.).

FISH with pan-CEN and CEN-specific probes has been performed according to the manufacturer's protocols. In sequential hybridization, sperm nuclei were first hybridized with pan-CEN, then subjected to several rounds of hybridization using combinations of chromosome-specific centromere probes. After each hybridization step, slides were mounted using DAPI/antifade and microscopic images were taken. To proceed to the next round of FISH, coverslips were gently removed; slides were rinsed in 0.1% SDS/0.2XSSC at 50°C, in 2XSSC at RT, and dehydrated/fixed in EtOH.

### Microscopy and image analysis

Microscopy was performed on a Leitz Ortholux fluorescent microscope using 63×, 1.4 NA oil objective and equipped with selective filters. Images were captured using a MagnaFire digital color camera and MicroFire software (Optronics Inc). Images were processed in Adobe Photoshop 7.0 and CS5 (Adobe Systems Inc.). To obtain the multicolor composite images after multistep sequential FISH, signals from individual CENs were selected with the “magic wand” option, artificial colors were assigned to the selected areas and images were merged.

**Figure 1:**
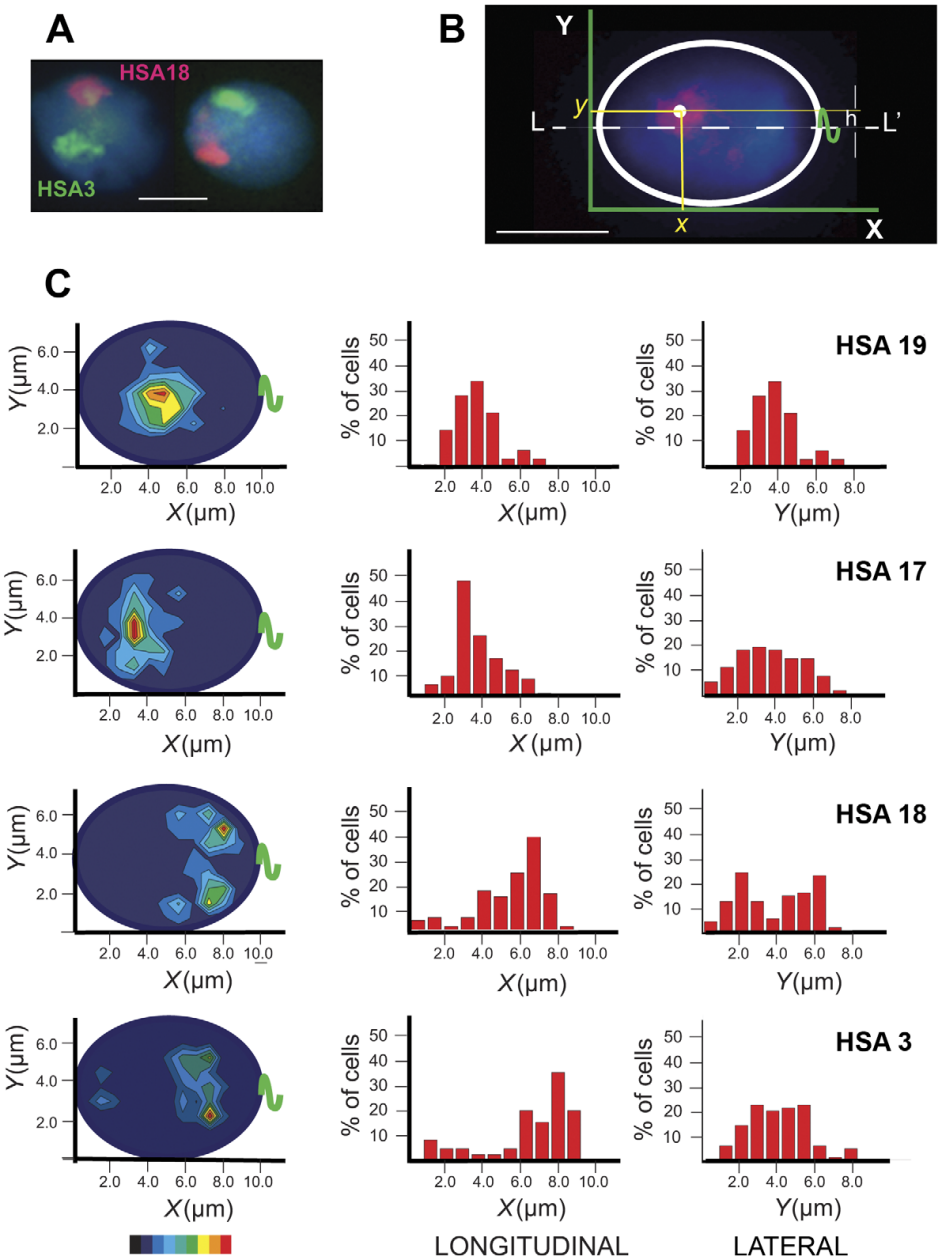
Localization of chromosome territories in human spermatozoa. (A) A typical image of chromosome territories in spermatozoa obtained using FISH with WCP probes. HSA18 paint – red, HSA3 – green, total DNA stained with DAPI – blue. (B) The scheme explaining the determination of CT center coordinates following FISH. The apical end of the ellipsoid sperm cell is on the left (***x*** = 0), the tail (green) attachment point - on the right. (C) Examples of the statistical evaluation of chromosome positioning. Position of each CT was determined in ≥80 cells. Left - contour plots showing the probability to find the CT center within the given area of the nucleus (red – the most probable localization). The color-coded bar at the bottom of the figure represents the p-value, with the red indicating p≤0.125 (the most probable localization) and the navy 0.875≤p≤1.000. The central and the right panels – frequency distribution plots for the longitudinal (along the long nuclear axis) and the lateral (along the short nuclear axis) positioning, respectively. Scale bar – 5 µm.

For CT localization measurements, nuclei with compact almost round WCP signals were selected. All measurements were made using Sigma Scan (Systat Software Inc.) according to the scheme outlined in Fig. 1B. To account for differences in nuclear swelling for individual cells, the measured values ***x*** and ***y*** were calibrated against the average size of swollen sperm nucleus in microns (10×7). At least 80 cells were examined for each chromosome. Histograms and contour plots were generated using Origin 8.6 (OriginLab Corp.) software. The preferable CT/peri-CEN position was determined using Gaussian approximation of frequency distribution plots. Data analysis was implemented using Origin 8.6 (OriginLab Corp.) and MS Excel 2010.

## Results and Discussion

### Preferred longitudinal and lateral intranuclear positioning of human CHRs in spermatozoa

Data indicating that CHRs in Hsp have non-random localization surfaced about 10 years ago [29], [30], [31] and were based on localization of peri-CEN sequences. Qualitative analysis using FISH with arm-specific or painting probes supported the existence of the preferred CHR positioning in spermatozoa [26], [32], [33]. In this study, we localized territories of eight CHRs along with their CENs in Hsp and applied a new semi-quantitative approach to the CHR positioning analysis. Territories of *Homo sapiens* chromosomes (HSA) 1, 3, 6, 17, 18, 19, X, and Y were visualized in the nuclei of Hsp using FISH with WCP probes (Fig. 1A).

Mature human spermatozoa are highly polarized cells with the acrosome located at the anterior pole of the elongated nucleus and the tail - at the posterior pole. The heads of human spermatozoa are flattened. Upon loading on microscope slide, they adopt one of the two most likely positions, one or other flat side down (similar to tossing a coin, when it comes up heads or tails). The shape of the sperm nucleus in this case can be approximated by an ellipse with the posterior part clearly detectable by the sperm tail preserved in the majority of cells under mild decondensation conditions (discussed earlier in [30], [32]).

Anteroposterior polarization of the flattened cell supplemented with a fixed spatial marker (tail attachment point) provides for the “natural” intrinsic 2D coordinate system and permits an unambiguous determination of the intranuclear location of the hybridization signals (Fig. 1B). The CHR position can be characterized by longitudinal (along the long nuclear axis) and lateral (distance from the long axis) coordinates.

Sperm head size increased to 1.5 times its original size, as judged by the long axis length, following DTT/Heparin treatment. The long to short axis ratio remained constant, equal to ∼1.4. This observation is in line with previous studies reporting the proportional increase in ***x***, ***y*** dimensions of sperm nuclei decondensed with DTT/Heparin [25], [26], [27].

For each CHR, the localization of the CT center was determined by measuring ***x*** (longitudinal) and ***y*** (lateral) coordinates followed by correction to the average ***x*** and ***y*** dimensions of a swollen sperm nucleus. Preferred longitudinal and lateral positions demonstrated in color-filled contour plots showing the probabilities of finding a given CT center within the defined area of a 2D ellipsoid which represented a sperm nucleus (Fig. 1C, left) and in frequency distribution histograms (Fig. 1C, center and right).

**Figure 2:**
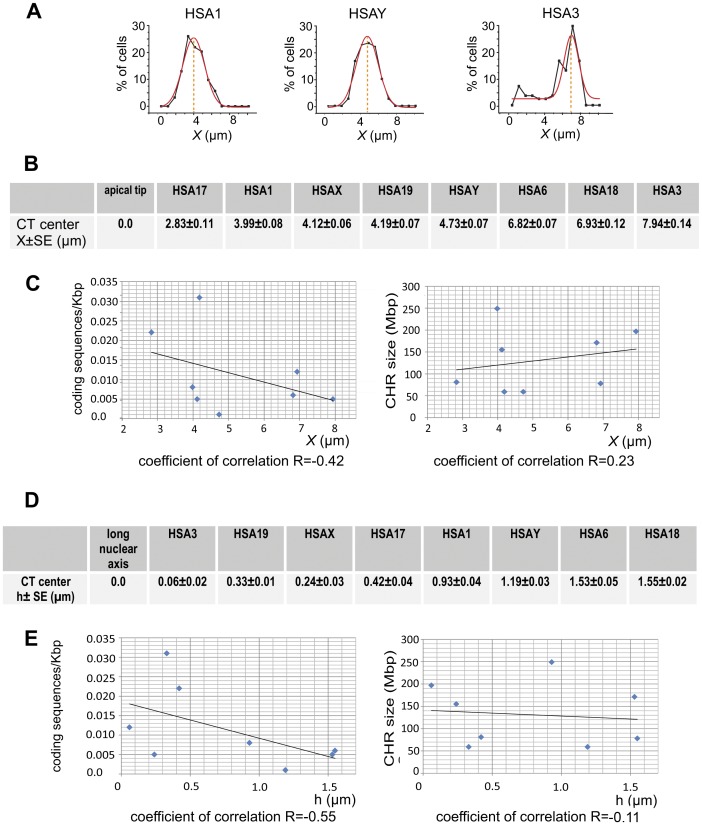
Relation between chromosome properties and their intranuclear localization in spermatozoa. (A) Examples of the CHR longitudinal coordinate determination using the Gaussian approximation (red line) of the frequency distribution data (black line). Numerical values of longitudinal (B) and lateral (D) coordinates of the CT centers. Correlations between the longitudinal (C) or the lateral (E) chromosome positioning and densities of coding sequences (left panels) or the chromosome size (right panels). ***x*** – the distance from the apical end of the sperm nuclei, ***h*** – the distance between the CT center and the long nuclear axis as described in the scheme Fig. 1,B.

Both contour plots and x-histograms (Fig. 1C, left and central panels, Fig. S1A) demonstrated an obvious preferential localization of CHRs along the nucleus length (longitudinal positioning), which is a novel sperm-specific feature indicated recently for humans [33] and demonstrated for other mammals with elongated spermatozoa [34], [35]. According to our data, HSA 1, 17, 19, X and Y tend to be in the anterior and HSA 3, 6, 18 - in the posterior half of sperm nuclei (Fig. S1A). The numerical values for the preferred ***x*** coordinates of CT centers (Fig. 2B) were determined using the Gaussian approximation of the frequency distribution graphs (Fig. 2A). The analysis of the longitudinal positioning in relation to CHR characteristics, such as gene density and size (Fig. 2C), demonstrated a negligible positive correlation between CHR remoteness from the apical end and CHR size (coefficient of correlation R = 0.23). The correlation between CHR position and gene richness was slightly higher and negative (R = −0.42) - specifically CHRs with higher density of protein coding sequences tended to occupy the apical end of spermatozoa.

Y histograms and contour plots showed the existence of non-random lateral positioning of CTs in spermatozoa (Fig. 1C and Fig. S1B). Depending on CT center position relative to the long nuclear axis (L-L′, scheme Fig. 1B), all CHRs could be divided into two groups according to their lateral localization pattern: i) CHRs which are positioned on or close to the long axis, for instance HSA19 and 17 (Fig. 1C), HSAX (Fig. S1B); such CHRs have a single peak of the preferred lateral localization, ii) CHRs which are distanced from the long nuclear axis, HSA3 and 18 (Fig. 1C), HSA1, 6, and Y (Fig. S1B); these ones display two almost symmetrical areas of the most probable localization. For the CHRs belonging to the second group, peaks of preferred positioning are roughly equidistant from the L-L′ (Fig. 1B) and have almost equal heights, i.e. probabilities to find a chromosome in either of these locations are equal. The symmetry of peaks around L-L′ axis can be explained by two possible depositions of the flattened Hsp cells onto microscope slides (flipping coin model).

The numerical value of the lateral CHR location (Fig. 2D) was determined by the distance between the CT center and the long nuclear axis L-L′ (h in the scheme Fig. 1B). Similarly to somatic cells, gene-rich CHRs in sperm nuclei tend to be located more internally than gene-poor ones. Compare, for example, lateral localization of HSA19 (0.031 protein coding sequences/kb) and HSA18 (0.006 sequences/kb), Figures 1C and 2D. The correlation coefficient between preferred lateral localization of eight CHRs studied and their gene richness is 0.6 (Fig. 2E). The qualitative relationship between the central/peripheral location and the gene content was reported for porcine [35] and human [33] sperm cells. Our data did not demonstrate a correlation between the CHR size and the lateral positioning (correlation coefficient −0.11, Fig. 2E), which is in disagreement with results of Manvelyan and co-authors reporting a strong positive link between CHR size and the distance of the CHR from the nuclear center [33]. The reason for the discrepancies may be due to differences in sperm cell processing for FISH and methods of data analysis. For example, Manvelyan and co-authors used a harsh cell fixation and a qualitative assessment of CT position in spherical 3D nuclei using a small number of cells (30 spermatozoa) from one individual [33].

### Longitudinal position of chromosome territories correlates with the position of corresponding CEN

**Figure 3:**
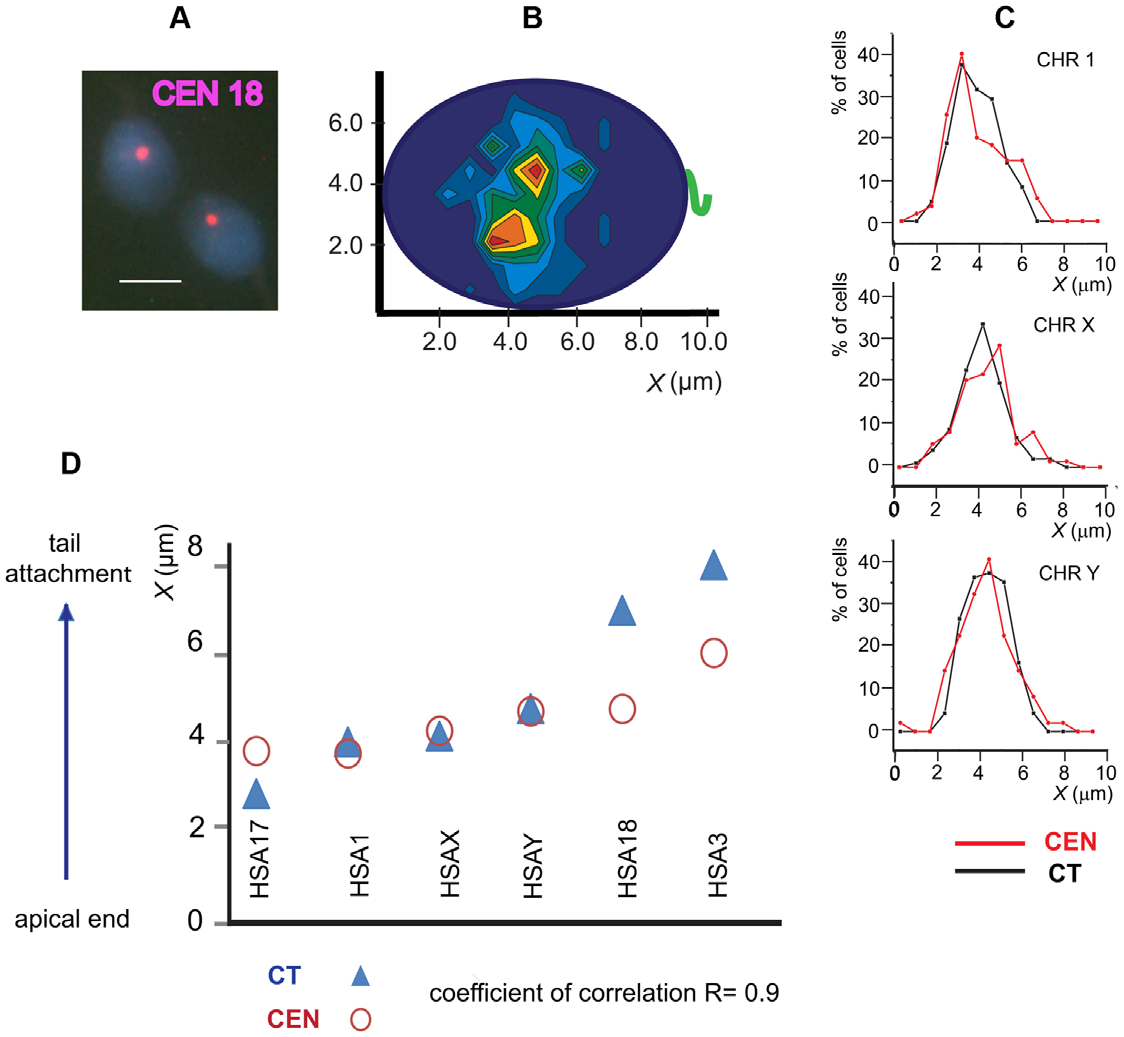
Localization of chromosome-specific peri-centromeric sequences in human spermatozoa. (A) Typical patterns of FISH with HSA18 peri-CEN probe. Scale bar – 5 µm. (B) The contour plot showing the preferential intranuclear localization of HSA18 (n≥80). (C) Sample frequency distribution plots for HSA 1, 17 and Y show that longitudinal localization of CTs (black lines) matches with the localization of corresponding CENs (red lines). (D) The correlation between the longitudinal positioning of CT and peri-CEN.

The size differences determined by the size of DNA sequence between CTs and CEN domains reach ∼200 times, and CTs in Hsp are characterized by conformation extended in anterior-posterior direction [26], [36]. Therefore, it is not clear *a priori* if and how longitudinal positioning of CTs relates with positioning of the corresponding CEN. To investigate this question, we have localized CHR-specific peri-CEN sequences of six CHRs - HSA 1, 3, 17, 18, Y, and X using FISH (Fig. 3A). Intranuclear positioning of CENs has been visualized using the procedure described above for the CTs, as illustrated for HSA18 in Fig. 3B. Comparison of the frequency distribution graphs (Fig. 3C) of the numerical values of ***x*** coordinates between the matching CENs and CTs (Fig. 3D) demonstrated a close similarity in their longitudinal positioning with a correlation coefficient R = 0.9. Starting from the apical end of the nucleus, both CTs and CENs of these six CHRs were located in the following sequence: 17→1→X→Y→18→3 (Fig. 3D). Preliminary data (not shown) indicate that this is also true for other chromosomes, i.e. HSA 19, 6, 4. The spread in the longitudinal localization of the most distant CTs (HSA17/HSA3) is ∼5 µm, while corresponding CENs are located much closer and separated by ∼2 µm – a tendency expected from the compactness of the Hsp chromocenter [25], [32]. Therefore, the evaluation of CT longitudinal positioning may be attained by localization of CEN domains, which is a less time consuming and a less complicated procedure. The comparison of the CT and CEN lateral localization demonstrated the absence of a link between these parameters, the correlation coefficient R = 0.14. Again, similarly to the ***x*** coordinate distribution, the spread of the ***y*** coordinate for CTs (2 µm) is larger than that for CENs (0.6 µm).

### CENs of nonhomologous CHRs are organized in arrays with a conserved linear order

**Figure 4:**
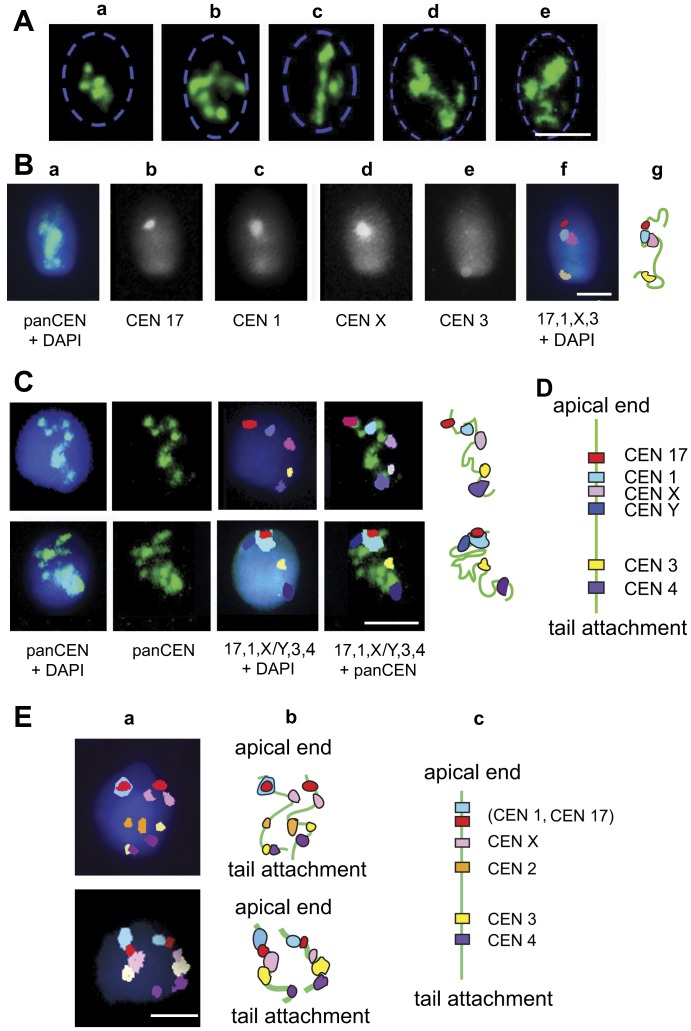
In human spermatozoa, nonhomologous centromeres are arranged in arrays with the fixed chromosome-specific linear order. (A) Visualization of CEN arrays using FISH with pan-CEN DNA probe. Nucleus borders, determined by DAPI staining are shown by a blue dashed line. (B) The outline of the sequential FISH procedure. First, cells were hybridized with pan-CEN probe (a, green). Cells that demonstrated unfolded CEN strings were subjected to sequential FISH with chromosome-specific peri-CEN probes (b–e). (f) - Artificial colors were assigned to the peri-CEN signals and images were merged. (g) - Schematic representation of the chromosome-specific peri-CEN localization. (C) Examples of CEN localization along sperm chromocenter arrays. (D) The cumulative scheme. (E) The order of CENs is preserved in diploid sperm nuclei. (a) Diploid sperm cells revealed using FISH with chromosome-specific peri-CEN probes; merged images after sequential FISH. (b) - Schematic representation of chromosome-specific peri-CEN localization. (c) - Cumulative scheme. Noteworthy, two sets of chromosomes have the same linear order matching with the arrangement established in haploid sperm nuclei (D). Scale bars in A–E – 5 µm.

Earlier studies of human sperm CENs using immunofluorescent localization of CENP-A and FISH with α-satellite DNA showed that CENs are clustered in a compact chromocenter [25], [27]. Upon an artificially induced nucleus swelling *in vitro*, this chromocenter was gradually dispersed, and CEN associations were observed at the intermediate stages of decondensation [25]. Here, we performed a more detailed evaluation of these structures using pan-CEN FISH: in ∼20% of Hsp, the chromocenter remained in a compact folded state (Fig. 4A a). More than 50% of the examined cells demonstrated uninterrupted CEN arrays folded, arched or almost linear (Fig. 4A b–e). The remaining ∼30% - had fragmented CEN arrays (not shown).

This allowed us to assess whether a defined order of CENs belonging to different CHRs exists within arrays. To address this, we used sequential FISH. The complete procedure consisted of the hybridization with pan-CEN probe which was followed by several rounds of FISH with CHR-specific peri-CEN probes (Fig. 4B). FISH results were recorded at all steps (Fig. 4B a–e). Only the spermatozoa demonstrating stretched (Fig. 4A b–e) and continuous arrays of centromeres (as judged by pan-CEN FISH) were selected for the concluding examination, in which CEN order along the strings was reconstructed using merged images (Fig. 4B f, g). A consistent linear order of CENs from the apical end of the nuclei to the tail attachment point 17→1→X→Y→3→4 repeated from cell to cell was registered (Fig. 4C, D), and this was observed for all examined spermatozoa (25 cells). Strikingly, this linear sequence of the CENs directly established in individual cells matched with the preferential longitudinal positioning of the CENs and CTs established by statistical analysis of the entire population of spermatozoa (compare Fig. 2B, 3C, 3D and 4D). The conservation of the favored CHR positioning demonstrated here is remarkable in the context of the long-lasting discussion about the existence and maintenance of the universal CHR order in somatic cells [6], [13], [14], [37]. Direct studies concerning the propagation of the global CHR positioning to the daughter cells conducted in live mammalian cells have not provided consistent conclusions [15], [16], [38], [39]. For example, it has been shown that the CHR order is maintained through mitosis resulting in strong similarities between mother and daughter cells [15]. A more recent study by Strickfaden et al. (2010), however, argues against the transmitting of chromosome positioning between cell generations and suggests the existence of the interphase mechanisms (such as rotational movements of CT assemblies) of specific CT proximity pattern formation starting with random CHR location in daughter cells [17].

The most intriguing questions concerning formation and maintenance of the observed CEN chains with the defined sequential order of elements that hypothetically define the preferred longitudinal localization of CTs in sperm nuclei are waiting for solutions. The physical link between CHRs has been observed in bovine or human capillary endothelial cells [40], [41] and in mouse pronuclei [42]. According to these studies, CHRs are interconnected with a participation of DNA and Topo II [40], [41], or by α-satellite DNA [42]. Occasional threads of CEN DNA between heterologous CHRs and pairing of nonhomologous CENs during the early stages of meiosis has been reported in evolutionarily distant organisms [43], [44]. Associations between heterochromatic regions, which are more pronounced in differentiated cells, are well-known for many organisms, including man [45], [46], [47], [48].

The regulation and molecular nature of coupling between CENs/CHRs has not been characterized yet. We speculate that the formation of chromocenter arrays in Hsp may be achieved through the α-satellite DNA linking via DNA catenation or by an association of CEN/peri-CEN domains through protein-protein interactions.

### Longitudinal chromosome order is set up at meiosis

The presence of a preferred longitudinal and lateral localization of CTs was shown by statistical analysis of the mixed population of sperm cells from three unrelated donors and by a direct cell to cell visualization. This suggests that CHR order is conserved within a sperm cell population in an ejaculate and possibly among individuals. It is important to know when in development and how this non-random CHR arrangement is established. Repositioning of CHRs X,Y and 13 during spermiogenesis in porcine [35] and sex CHRs in mouse [49] has been observed. In differentiating spermatids of these animals, CHR X migrates from the periphery to the center of the nucleus. Such repositioning could reflect the global rearrangement of chromatin/CHR during spermiogenesis and it may not affect the preferential longitudinal localization. A study of CHR distribution during human spermatogenesis has not been performed yet.

About 0.2% of sperm cells in the fertile human male are estimated to be diploid [50]. Indeed, during experiments exploring linear order of CHRs, we registered five morphologically normal diploid sperm cells as was evidenced by FISH with CHR-specific peri-CEN probes (Fig. 4E a, b). Remarkably, the linear order of CENs was identical in two genome sets of diploid sperm cells and essentially matched the established longitudinal arrangement of CHRs in haploid sperm cells (compare data of Figs. 2B, 3D and 4D). Based on this observation, we propose that the non-random and conserved longitudinal order of the CHRs characteristic for the elongated Hsp is established during the meiotic stages of spermatogenesis.

Our data indicate an apparent origination of the favored relative CHR positioning characteristic to spermatozoa in meiotic cells or the permanent existence of such. Nagele and co-authors proposed the existence of two spatially distinct orderly positioned CHR sets inherited from each parent at the time of fertilization [13], but this hypothesis has not been confirmed so far.

### Refined model of CHR organization in human spermatozoa and it implications for fertilization

**Figure 5:**
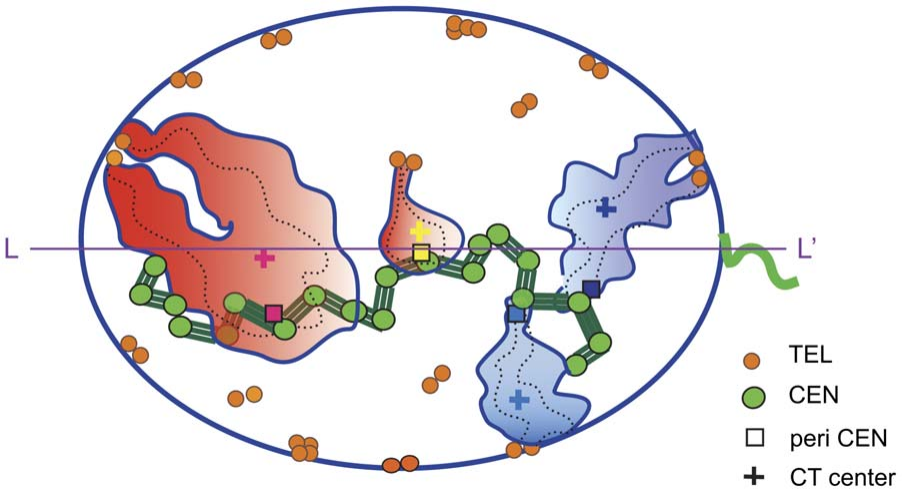
Model of chromosome organization in human spermatozoa. Compact CTs (filled contours) have overall hairpin conformations (chromosome paths indicated by dashed lines) with the *p* and *q* telomere/sub-telomere domains (orange circles) forming dimers at nuclear periphery. Gene-rich CHRs – rosy, gene-poor – indigo. CTs are connected via centromeres/peri-centromeres (green circles and lines) into arrays and have a fixed linear order which determines the longitudinal positioning of chromosomes.

Spatial arrangement and architecture of CHRs in human sperm nuclei is much more ordered than in somatic cells, probably because sperm cells are genetically inert and their DNA is super-condensed by protamines [22], [51]. In Hsp, CTs have an extended shape [26], [27], [36] and hairpin-like conformation with telomeres of *p-* and *q-* arms forming dimers at the nuclear periphery [26], [52]. In the extension of our earlier observations [25], we demonstrated that CENs (Fig. 5, green circles) form continuous arrays (Fig. 5, green lines), indicating that sperm CHRs may be physically connected, possibly via CEN/peri-CEN chromatin.

This work and the previous ones [32], [33] demonstrate a pronounced certainty in the intranuclear localization of CHRs in sperm. Such a preferred positioning has been established by localization of CHR-specific peri-CEN domains (Fig. 5, filled squares) or CT centers (Fig. 5, crosses). In the 2-D representation of the elongated human sperm nucleus, the CHR localization has the “longitudinal” and the “lateral” components. We established that ***x*** coordinates of either CT centers or peri-CENs are tightly linked while the latter are orderly placed along the chromocenter array (green lines in Fig. 5). Lateral positioning of the CT centers and CENs are not correlated.

To outline the relation between sperm cell CHR positioning and their gene content, two archetypal gene-rich CHRs (Fig. 5B, red territories) and two gene-poor CHRs (Fig. 5B, blue territories) are shown. According to our data gene-rich CHRs tend to localize closer to the apical end and preferably in the interior part of the nucleus. In contrast, gene-poor chromosomes were found closer to the periphery and the basal area of the nucleus. This is a new (apparently functional) feature of the emerging model that describes sperm nuclear organization. A second novel characteristic is the link between the longitudinal localization of CTs and corresponding centromere domains. In turn, CEN linear positioning is dictated by chromocenter arrays which may be supported by catenation of CEN DNA, promoted by TOPOII, or/and protein-protein interactions provided by CEN-B. Identification of the molecules contributing to CEN connections is significant for understanding the interactions between non-homologous CHRs in different human cell types including gametes.

Emerging deliberate arrangement of CHRs in human sperm nuclei could imply its significance in early fertilization events. It has been hypothesized that at fertilization, a differential spatial-temporal exposure of sperm CHRs and selected chromosomal domains to components of the ooplasm takes place [24]. Molecules of the ooplasm induce an uneven sperm chromatin remodeling and commence transcription, which results in programmed activation of the male genome. Proximity of sperm CHRs to the nucleus periphery or to the point of the first contact of the sperm nucleus with the ooplasm was suggested to be the epigenetic basis for differential transcription and replication during early embryogenesis [24], [34], [35]. In addition, a specific arrangement of CHRs in sperm nuclei may participate in setting up chromosome organization in the embryo. Noteworthy, an aberrant CHR positioning has been observed in sperm cells from some infertile individuals [53], [54].

The further detailed examination of the intranuclear localization of the paternal CHR set in sperm nuclei and during the pronuclei formation in the early zygote is under investigation.

## Supporting Information

Figure S1 Frequency distribution plots for the longitudinal and the lateral positioning of the eight CHRs in human spermatozoa. Statistically preferred lateral coordinates of chromosomes detached from the long nuclear axis demonstrate two symmetrical peaks.
(TIF)
