# Supplementary figures and images for: Positioning of Chromosomes in Human Spermatozoa Is Determined by Ordered Centromere Arrangement
Source: PLoS One. 2012 Dec 27;7(12):e52944. doi: 10.1371/journal.pone.0052944 (PMC3531364; doi:10.1371/journal.pone.0052944)

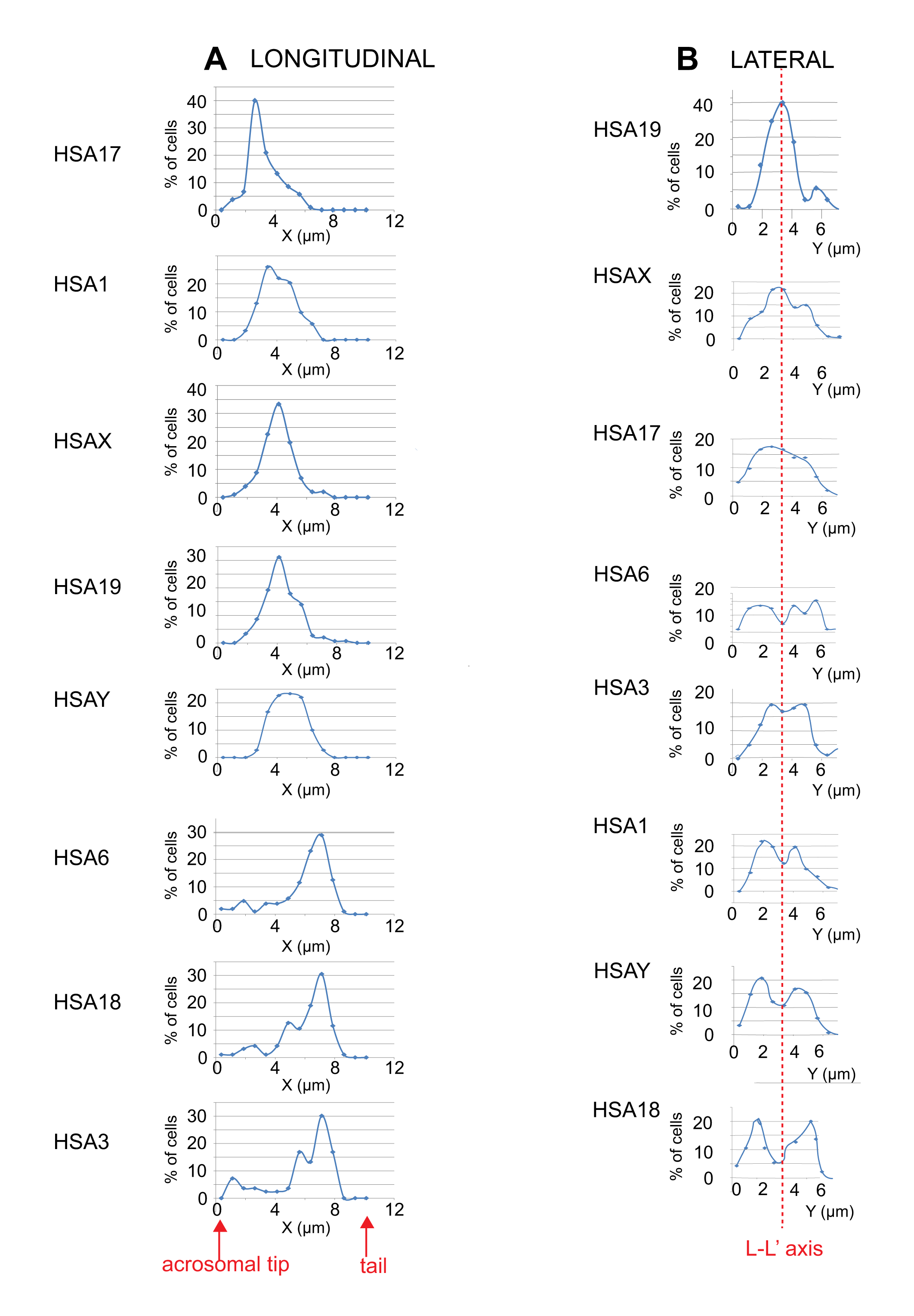

Supplement: Figure S1 — Frequency distribution plots for the longitudinal and the lateral positioning of the eight CHRs in human spermatozoa. Statistically preferred lateral coordinates of chromosomes detached from the long nuclear axis demonstrate two symmetrical peaks. (TIF) [file pone.0052944.s001.tif]
